# Real‐World Insights From Türkiye: Biologic DMARDs Usage in Spondyloarthritis Patients With Chronic Kidney Disease

**DOI:** 10.1111/1756-185X.70274

**Published:** 2025-05-13

**Authors:** Dilara Bulut Gökten, Mehmet Engin Tezcan, Burcu Yağız, Abdulsamet Erden, Gezmiş Kimyon, Nazife Şule Yaşar Bilge, Levent Kılıç, Belkıs Nihan Coşkun, Emine Duygu Ersözlü, Orhan Küçükşahin, Süleyman Serdar Koca, Emel Gönüllü, Muhammet Çınar, Servet Akar, Hakan Emmungil, Timuçin Kaşifoğlu, Cemal Bes, Aşkın Ateş, Yavuz Pehlivan, Sedat Kiraz, Ali İhsan Ertenli, Hüseyin Ediz Dalkılıç, Umut Kalyoncu, Rıdvan Mercan

**Affiliations:** ^1^ Division of Rheumatology, Department of Internal Medicine Tekirdag Namik Kemal University Tekirdag Türkiye; ^2^ Division of Rheumatology, Department of Internal Medicine Kartal Dr. Lütfi Kırdar City Hospital Istanbul Türkiye; ^3^ Division of Rheumatology, Department of Internal Medicine Faculty of Medicine, Bursa Uludag University Bursa Türkiye; ^4^ Division of Rheumatology, Department of Internal Medicine Gazi University Ankara Türkiye; ^5^ Division of Rheumatology, Department of Internal Medicine Hatay Mustafa Kemal University Hatay Türkiye; ^6^ Division of Rheumatology, Department of Internal Medicine Eskişehir Osmangazi University Eskişehir Türkiye; ^7^ Division of Rheumatology, Department of Internal Medicine Hacettepe University Ankara Türkiye; ^8^ Division of Rheumatology, Department of Internal Medicine Adana City Research and Training Hospital Adana Türkiye; ^9^ Division of Rheumatology, Department of Internal Medicine Yildirim Beyazit University Ankara Türkiye; ^10^ Division of Rheumatology, Department of Internal Medicine Firat University Elazig Türkiye; ^11^ Division of Rheumatology, Department of Internal Medicine Sakarya University Sakarya Türkiye; ^12^ Division of Rheumatology, Department of Internal Medicine Gülhane Training and Research Hospital Ankara Türkiye; ^13^ Division of Rheumatology, Department of Internal Medicine Izmir Katip Çelebi University Izmir Türkiye; ^14^ Division of Rheumatology, Department of Internal Medicine Trakya University Edirne Türkiye; ^15^ Division of Rheumatology, Department of Internal Medicine Istanbul Basaksehir Cam and Sakura Hospital Istanbul Türkiye; ^16^ Division of Rheumatology, Department of Internal Medicine Ankara University Ankara Türkiye

**Keywords:** adverse event, biologic DMARDs, chronic kidney disease, safe use, spondylarthritis

## Abstract

**Aim:**

The objective was to evaluate biologic disease‐modifying antirheumatic drugs (DMARDs) and their side effects that hindered the continuation of treatment in a patient population diagnosed with spondyloarthritis (SpA) with a glomerular filtration rate (GFR) ≤ 60 mL/min, and to compare these side effects between patients with chronic kidney disease (CKD) and those without.

**Methods:**

This multicenter, observational cohort study utilized data from the TReasure database, which records SpA patients in a web‐based system across Türkiye. A total of 6052 patients being included. SpA patients were categorized into two main groups: non‐CKD patients and CKD patients. The clinical characteristics, disease activity, treatment options, drug retention rates, reasons for drug discontinuation, and types of adverse effects were compared between the groups.

**Results:**

Biologics prescription pattern varied between CKD and non‐CKD patients. Etanercept was prescribed more frequently (53.1%) in CKD patients. Regarding the number of side effects and drug discontinuations in CKD patients, no statistically significant differences were found between the non‐CKD and CKD groups for any of the bDMARDs (adalimumab, etanercept, golimumab, infliximab, ustekinumab, secukinumab, and certolizumab). No statistically significant differences were observed in the duration of drug retention based on CKD status for bDMARDs.

**Conclusion:**

This study offers preliminary evidence supporting the effective and safe use of bDMARDs in patients with SpA and CKD.

## 1 Introduction

Chronic kidney disease is a progressive disorder impacting over 10% of the global population, affecting more than 800 million people worldwide [1]. Chronic kidney disease (CKD) is defined as either structural or functional kidney damage, or a sustained reduction in glomerular filtration rate (GFR) below 60 mL/min/1.73 m^2^ for a duration of at least 3 months, irrespective of the underlying cause [2]. Kidney damage in various renal conditions is commonly detected by the presence of albuminuria, defined as an albumin‐to‐creatinine ratio greater than 30 mg/g in at least two out of three spot urine samples. Most patients with CKD fall into grade G3, characterized by an eGFR of 30–59 mL/min/1.73 m^2^ [3].

The kidneys play a crucial role in eliminating numerous drugs and metabolites from the human body [4]. Renal dysfunction can markedly alter the pharmacokinetics and pharmacodynamics of many medications, potentially compromising their safety, efficacy, and overall risk–benefit profile [5]. As a result, dose adjustments are often required, and certain drugs may need to be avoided in patients with CKD [6].

Spondyloarthritis (SpA), also referred to as spondyloarthropathy, serves as an overarching term for a cluster of diseases. Notable conditions within this category include ankylosing spondylitis (AS), psoriatic arthritis (PsA), reactive arthritis (ReA), spondyloarthritis associated with inflammatory bowel disease (IBD), and undifferentiated forms of spondyloarthritis [7]. Although the incidence of SpA varies across different regions globally, it is generally regarded as a common rheumatic condition, affecting approximately 0.5%–2% of the population [8]. Studies in Türkiye reveal an adjusted SpA prevalence of 0.46%, with the highest prevalence of 1.28% observed in the 25–34 age group [9]. In Türkiye, biologic therapies are covered by the national healthcare system but are subject to strict reimbursement regulations. The Social Security Institution (SGK) typically requires prior failure of conventional DMARDs, and some biologics are restricted to specific indications or treatment steps. These constraints may influence clinical decision‐making and real‐life treatment patterns.

The optimal pharmacological approach for managing patients with both CKD and SpA remains a subject of debate. To date, only a limited number of studies have investigated the use and pharmacokinetics of biologic antirheumatic agents in the context of CKD [10]. The objective of this study was to assess the use of biologic DMARDs and the side effects leading to treatment discontinuation in patients with various subtypes of SpA who had CKD (GFR ≤ 60 mL/min), and to compare these findings with those in SpA patients without CKD.

## 2 Material and Methods

### 2.1 The Selection and Classification of Patients Based on Renal Function

Established in 2017, the TReasure Registry is a national web‐based platform in Türkiye, designed to longitudinally monitor RA and SpA patients undergoing treatment with bDMARDs or tsDMARDs. This multicenter, observational cohort study utilized data from the TReasure database, which records SpA patients in a web‐based system across 15 centers in Türkiye [11]. There were a total of 7157 patients aged 18 years or older diagnosed with SpA. Data from 1105 individuals were incomplete, and some were later diagnosed with CKD. Patients with incomplete demographic data, laboratory results, or clinical visit notes, as well as those who developed CKD after starting biologic therapy, were excluded, resulting in a total of 6052 patients being included. The TReasure registry applied several classification criteria for diagnosing SpA, including the modified New York criteria [12], the 2009 EULAR criteria for axial and peripheral SpA [13], the Assessment of SpondyloArthritis International Society (ASAS) criteria for non‐radiographic axial SpA, and the CASPAR (Classification of Psoriatic Arthritis) criteria [14, 15]. SpA patients were categorized into two main groups: non‐CKD patients (*n* = 5892) and CKD patients (*n* = 160). To assess renal function, CKD was defined according to the Kidney Disease: Improving Global Outcomes (KDIGO) 2012 guideline [16]. The glomerular filtration rate (GFR) was estimated using the modified Modification of Diet in Renal Disease (MDRD) formula [17].

### 2.2 Clinical and Laboratory Assessment of Patients and Evaluation of Outcome Measures

The study evaluated various demographic, clinical, and laboratory characteristics, including age, sex, disease duration, and treatments administered prior to the initiation of biologic therapy. Additional parameters such as comorbidities, use of conventional synthetic DMARDs (methotrexate, sulfasalazine, leflunomide) at baseline and during follow‐up, smoking history, ESR (mm/h), CRP (mg/L), HLA‐B27 status, patterns of biologics utilization (both ever and first‐line), drug discontinuation due to adverse events (including reasons such as social security issues, pregnancy planning, and physician or patient request), the number and types of adverse events, and drug retention duration according to CKD status were also analyzed. The reasons for switching or discontinuing bDMARDs were categorized as follows: ineffectiveness (primary or secondary failure, as determined by the rheumatologist), adverse events, and non‐adverse events (e.g., patient or doctor preferences). The bDMARDs administered to all patients included anti‐tumor necrosis factor (TNF) agents, as well as anti‐IL17, IL12/IL23 inhibitors including etanercept, infliximab, golimumab, adalimumab, certolizumab, secukinumab, and ustekinumab. Outcome measures included Bath AS disease activity index (BASDAI), Bath AS functional index (BASFI), AS disease activity score with CRP (ASDAS‐CRP), visual analogue scale (VAS) assessments by both the physician and the patient (VAS‐PGA and VAS‐DGA; 0–100 mm), health assessment questionnaire‐disability (HAQ) index, and Charlson comorbidity index (CCI) scores.

### 2.3 Statistical Analysis

Statistical analyses were performed using PASW Statistics 18.0 for Windows. Descriptive statistics were expressed as counts and percentages for categorical variables, and as mean, standard deviation, median, 25th percentile, and 75th percentile for continuous variables. The normality of data distribution was evaluated using both visual (histograms and probability plots) and analytical methods (Kolmogorov–Smirnov and Shapiro–Wilk tests). For comparisons of categorical variables, the Chi‐square test was applied when assumptions were met; otherwise, Fisher's Exact Test or the Extended Fisher's Exact Test was used. For continuous variables not meeting the assumption of normality, the Mann–Whitney U test was employed for comparisons between two groups. A *p* value < 0.05 was considered statistically significant.

## 3 Results

### 3.1 Demographical Data, Comorbidities and Outcome Measures

In this study, CKD was diagnosed in 160 of the 6052 patients. CKD patients were significantly older and had a higher mean BMI, as well as a longer mean disease duration (*p* < 0.001), compared to those without CKD. Although the gender distribution, smoking history, HLA‐B27 positivity, retention time on the first bDMARD, and the time from symptom onset to diagnosis were similar between the groups, the treatment duration before initiating biologic therapy was notably longer in patients with CKD.

Amyloidosis, hypertension, diabetes mellitus, hyperlipidemia, and coronary artery disease were significantly more common in CKD patients, with amyloidosis observed in 10% of these patients compared to a lower percentage in non‐CKD patients (*p* < 0.001). Regarding disease subtypes, AS, peripheral SpA, and enteropathic SpA were observed at similar rates between CKD and non‐CKD patients, while PsA was more frequent in CKD patients (*p* = 0.003) and nr‐axSpA was less common (*p* = 0.001). Clinically, psoriasis and arthritis were more prevalent in CKD patients (*p* = 0.001 and *p* = 0.003), whereas dactylitis and enthesitis rates were comparable between the groups.

CRP and ESR levels were significantly elevated in patients with CKD (*p* = 0.042 and *p* < 0.001, respectively). No significant differences were observed between the groups in terms of ASDAS‐CRP, HAQ, BASDAI, or BASFI scores. The Charlson Comorbidity Index (CCI) was significantly higher in the CKD group (*p* < 0.001).

### 3.2 Medication Utilization Patterns Between Groups

Prior to the initiation of bDMARD therapy, the use of steroids and leflunomide was significantly more frequent among CKD patients (*p* = 0.004 and *p* = 0.001, respectively), whereas the use of NSAIDs and sulfasalazine was significantly lower in this group (*p* = 0.001 and *p* = 0.038). Methotrexate use did not differ significantly between the groups (*p* = 0.082) (see Table 1).

**TABLE 1:** Demographic characteristics and drug treatments of patients before initiating biologic therapy.

Demographical data and characteristics of patients			
	CKD patients, *n*: 160	Non‐CKD patients, *n*: 5892	*p*
Age, years	58 ± 11	44 ± 11	***< 0.001***
Gender (F/M) (%)	42.5/57.5	45.6/54.4	0.434
Disease duration (years) mean ± SD	12.95 ± 10.69	8.45 ± 7.81	***< 0.001***
Duration of treatment (months) mean ± SD	81.00 ± 58.81	61.70 ± 49.60	***< 0.001***
Duration of persistence with the first bDMARD (months), mean ± SD	46.29 ± 48.13	37.94 ± 40.32	0.158
Time from symptom onset to diagnosis (years), mean ± SD	5.07 ± 6.67	4.35 ± 5.98	0.327
BMI, mean ± SD	29.65 ± 5.61	27.40 ± 5.31	***< 0.001***
Smokers (ever), %	57.4	58.9	0.709
HLA‐B27 positivity %	43.0	49.1	0.262
Disease subtypes			
As, *n* (%)	92 (57.5)	3620 (61.4)	0.941
Nr‐Ax SpA, *n* (%)	3 (1.8)	592 (10.0)	***0.001***
PsA, *n* (%)	30 (18.7)	560 (10)	***0.003***
Peripheral SpA, *n* (%)	30 (18.7)	803 (13.6)	0.897
Enteropathic arthritis, *n* (%)	5 (3.1)	317 (5.4)	0.210
Outcome measures			
BASDAI, mean ± SD	5.51 ± 2.17	5.66 ± 2.05	0.738
BASFI, mean ± SD	4.88 ± 2.26	4.57 ± 2.27	0.160
ASDAS‐CRP, mean ± SD	3.65 ± 0.97	3.54 ± 0.96	0.182
Pain (VAS)	67.01 ± 25.51	69.30 ± 23.25	0.794
VAS global, mean ± SD	67.02 ± 22.40	67.01 ± 21.63	0.821
VAS physician, mean ± SD	66.83 ± 21.07	61.36 ± 21.53	***0.012***
Fatigue (VAS)	61.14 ± 25.92	62.53 ± 25.61	0.683
CRP (mg/L), median	23.80 ± 31.87	20.53 ± 28.99	***0.042***
ESR (mm/h), median	36.24 ± 23.77	27.17 ± 22.08	***< 0.001***
HAQ Score, mean ± SD	0.76 ± 0.49	0.68 ± 0.48	0.053
CCI, mean ± SD	2.23 ± 1.19	1.17 ± 0.47	***< 0.001***
Comorbidity			
Amyloidosis, *n* (%)	15 (10.0)	23 (0.4)	***< 0.001***
Hypertension, *n* (%)	98 (61.6)	935 (15.9)	***< 0.001***
Diabetes mellitus, *n* (%)	37 (23.4)	463 (7.9)	***< 0.001***
Hyperlipidemia, *n* (%)	51 (33.3)	692 (12.9)	***< 0.001***
Coronary heart disease, *n* (%)	23 (14.6)	252 (4.3)	***< 0.001***
Extra‐articular manifestations			
Uveitis, *n* (%)	19 (11.9)	643 (10.9)	0.683
IBH, *n* (%)	5 (3.1)	317 (5.4)	0.209
Psoriasis, *n* (%)	46 (28.9)	1068 (18.1)	***0.001***
Arthritis, *n* (%)	47 (29.6)	1176 (20.1)	***0.003***
Dactylitis, *n* (%)	12 (7.5)	368 (6.3)	0.511
Enthesitis, *n* (%)	27 (17.1)	1175 (20.1)	0.358
Treatments (ever)			
Steroids, *n* (%)	22 (13.8)	450 (7.6)	***0.004***
NSAID, *n* (%)	79 (49.4)	4249 (72.1)	***0.001***
SSZ, *n* (%)	84 (52.5)	3573 (60.6)	***0.038***
MTX, *n* (%)	64 (40.0)	1969 (33.4)	0.082
Leflunomide, *n* (%)	31 (19.4)	549 (9.3)	***0.001***

*Note:* Chi‐square test, Mann–Whitney U test, Fisher exact test; *p* < 0.05 was shown as bold.

Abbreviations: AS, Ankylosing Spondylitis; ASDAS‐CRP, Ankylosing Spondylitis Disease Activity Score‐C‐Reactive Protein; BASDAI, Bath Ankylosing Spondylitis Disease Activity Index; BASFI, Bath Ankylosing Spondylitis Functional Index; bDMARD, Biological Disease‐Modifying Antirheumatic Drug; BMI, Body Mass Index; CCI, Charlson Comorbidity Index; CKD, Chronic Kidney Disease; CRP, C‐Reactive Protein; ESR, Erythrocyte Sedimentation Rate; HAQ, Health Assessment Questionnaire; HLA‐B27, Human Leukocyte Antigen B27; IBH, Inflammatory Bowel Disease‐Associated Arthritis; MTX, Methotrexate; Nr‐Ax‐SpA, Non‐radiographic Axial Spondyloarthritis; NSAID, Nonsteroidal Anti‐Inflammatory Drug; PsA, Psoriatic Arthritis; SpA, Spondyloarthritis; SSZ, Sulfasalazine; VAS, Visual Analog Scale.

The ever biological treatment prescription patterns of rheumatologists varied between CKD and non‐CKD patients. Adalimumab, infliximab, and golimumab were prescribed at similar rates in both groups. Etanercept was significantly more commonly used in CKD patients (53.1% vs. 34.0%, *p* < 0.001), while certolizumab was less commonly prescribed (*p* = 0.003). The use of ustekinumab (3.1%) and secukinumab (18.8%) showed no statistically significant differences between the groups.

Rheumatologists' first‐line patterns of biologics utilization showed distinct patterns as well. Adalimumab was less commonly prescribed to CKD patients (27.5% vs. 37.6%, *p* = 0.009), while etanercept was significantly more common in CKD patients (39.4% vs. 20.2%, *p* < 0.001). Infliximab and golimumab were used at similar rates between the groups. Certolizumab, however, was significantly less frequently used in CKD patients (3.8% vs. 11.3%, *p* = 0.003). Ustekinumab and secukinumab were infrequently prescribed in both groups, with no statistically significant differences observed (see Table 2).

**TABLE 2:** Biological treatment (ever) and first‐line biological treatment prescription patterns of rheumatologists according to kidney disease status of patients.

Biological treatment prescription patterns of rheumatologists (Ever)	CKD patients, *n*: 160	Non‐ CKD patients, *n*: 5892	*p*
Adalimumab	71 (46.9)	3041 (53.3)	0.107
Etanercept	81 (53.1)	2004 (34.0)	***< 0.001***
Infliximab	44 (28.1)	1427 (24.2)	0.256
Golimumab	27 (16.9)	1018 (17.3)	0.894
Certolizumab	24 (15.0)	1436 (25.2)	***0.003***
Ustekinumab	5 (3.1)	113 (1.9)	0.243
Secukinumab	25 (18.8)	799 (14.5)	0.131
First‐line biological treatment prescription patterns of rheumatologists			
Adalimumab	44 (27.5)	2214 (37.6)	***0.009***
Etanercept	63 (39.4)	1193 (20.2)	***< 0.001***
Infliximab	29 (18.1)	961 (16.3)	0.540
Golimumab	10 (6.3)	652 (11.1)	0.054
Certolizumab	6 (3.8)	663 (11.3)	***0.003***
Ustekinumab	1 (0.6)	10 (0.2)	0.255
Secukinumab	7 (4.4)	196 (3.3)	0.467

*Note:* Chi‐square and Fisher exact test. *p* < 0.05 was shown as bold.

Abbreviation: CKD, Chronic kidney disease.

### 3.3 Number and Types of Side Effects Leading to Drug Discontinuations in CKD Patients

Adalimumab was prescribed in 71 patients with CKD, resulting in 2 adverse events, while etanercept was used in 81 patients, with 13 adverse events reported. Golimumab was prescribed in 27 cases and led to 2 adverse events, whereas infliximab was used in 44 cases, resulting in 3 adverse events (6.8%). No statistically significant differences in adverse event rates were observed between CKD and non‐CKD groups for any of the biologic agents. Among non‐CKD patients, 859 out of 5892 individuals (8.7%) experienced adverse events that led to drug discontinuation, compared to 21 out of 160 patients (13.1%) in the CKD group. Allergies were reported in 198 non‐CKD patients (23%) and 1 CKD patient, with a statistically significant difference (*p* = 0.027). Injection site and infusion reactions were observed in 117 non‐CKD patients and in 3 patients with CKD. Non‐psoriatic skin lesions were not reported in any CKD patients. Psoriasis was documented in 4.7% of CKD patients, and infections were reported in 14%. However, none of these outcomes showed statistically significant differences between the CKD and non‐CKD groups (see Table 3).

**TABLE 3:** Number and types of side effects leading to drug discontinuations according to bDMARDs in CKD patients.

Side effects	*n*	CKD (eGFR < 60 mL/min/1.73 m^2^), *n* (%)	*n*	Non‐CKD (eGFR ≥ 60 mL/min/1.73 m^2^), *n* (%)	*p*
Adalimumab	71	2 (2.8)	3041	243 (7.9)	0.153
Etanercept	81	13 (16.0)	2004	206 (10.3)	0.751
Golimumab	27	2 (7.4)	1018	51 (5)	0.303
Infliximab	44	3 (6.8)	1427	216 (15.1)	0.689
Secukinumab	25	0 (0.0)	799	40 (5)	0.234
Certolizumab	24	1 (4.2)	1436	103 (7.2)	0.326

	CKD patients, *n*: 160, *n* (%)		Non‐CKD, *n*: 5892, *n* (%)		*p*
Side effects leading to drug discontinuation—no. (%)	21 (13.1)		859 (8.7)		0.508
Allergy	1 (4.7)		198 (23)		***0.027***
Injection site and infusion reaction	3 (14.2)		117 (13.6)		0.682
Skin lesions other than psoriasis	0 (0.0)		87 (10.1)		0.175
Psoriasis development	1 (4.7)		72 (8.3)		0.370
Infection	3 (14)		65 (7.5)		0.511
Uveitis development	3 (14)		50 (5.8)		0.127
GIS side effects	0 (0.0)		39 (4.5)		0.404
Shortness of Breath	2 (9.5)		36 (4.1)		0.342
Cancer or suspected cancer	3 (14.2)		28 (3.2)		0.076
Cardiovascular side effects	1 (4.7)		28 (3.2)		0.577
Liver function test deterioration	1 (4.7)		27 (3.1)		1.000
Neurologic side effects	0 (0.0)		20 (2.3)		1.000
Tuberculosis	0 (0.0)		20 (2.3)		1.000
IBH development	1 (4.7)		15 (1.7)		0.388
Lupus development	1 (4.7)		12 (1.3)		0.329
Leucopenia	0 (0.0)		8 (0.9)		1.000
Thrombocytopenia	0 (0.0)		4 (0.4)		—
Anemia	0 (0.0)		3 (0.3)		—
Hearing loss	0 (0.0)		4 (0.4)		—
Proteinuria	0 (0.0)		4 (0.4)		1.000
Zoster infection	0 (0.0)		4 (0.4)		—
Sarcoidosis	0 (0.0)		4 (0.4)		—
Vasculitis, Raynaud phenomenon	0 (0.0)		5 (0.58)		1.000
Hair loss	1 (4.7)		5 (0.58)		0.142
Headache	0 (0.0)		4 (0.4)		—

*Note:* Fisher's Exact Test, Chi‐square test; *p* < 0.05 was shown as bold; (*n*: ever use).

Abbreviations: CKD, chronic kidney disease; GIS, Gastrointestinal System; IBH, Inflammatory Bowel Disease‐Associated Arthritis.

### 3.4 Duration of Drug Retention Based on CKD Status

The duration of drug retention was analyzed based on CKD status. For adalimumab, etanercept, golimumab, infliximab, secukinumab, certolizumab, and ustekinumab, retention times were comparable between the groups (*p* = 0.055, 0.224, 0.727, 0.602, 0.932, 0.222, and 0.211) (see Table 4).

**TABLE 4:** Drug retention duration (months) based on CKD status.

	Non‐CKD patients (eGFR ≥ 60 mL/min/1.73 m^2^) mean ± SD (Min–Max)	CKD patients (eGFR < 60 mL/min/1.73 m^2^) mean ± SD (Min–Max)	*p*
Adalimumab	20.72 ± 24.42 (0.10–159.11)	30.46 ± 34.01 (3.06–135.82)	0.055
Etanercept	28.32 ± 32.11 (0.10–249.56)	35.16 ± 36.24) (1.77–129.31)	0.224
Golimumab	20.93 ± 21.06 (0.03–112.26)	22.43 ± 19.89 (2.37–62.39)	0.727
Infliximab	29.36 ± 31.67) (0.03–181.91)	30.48 ± 34.59 (0.07–128.10)	0.602
Secukinumab	10.56 ± 9.72) (0.10–59.99)	12.98 ± 11.63 (2.10–28.45)	0.932
Certolizumab	13.91 ± 14.66 (0.20–124.35)	21.06 ± 16.51 (2.60–47.34)	0.222
Ustekinumab	15.60 ± 18.86 (1.05–100.01)	16.62 ± 6.32 (9.66–21.98)	0.211
All‐TNF inhibitors	22.64 ± 24.784 (0.03–249.56)	27.91 ± 28.248 (0.07–135.82)	0.095

*Note:* Mann–Whitney U test; *p* < 0.05 was shown as bold.

Abbreviations: CKD, Chronic kidney disease; SD, standard deviation.

**FIGURE 1:**
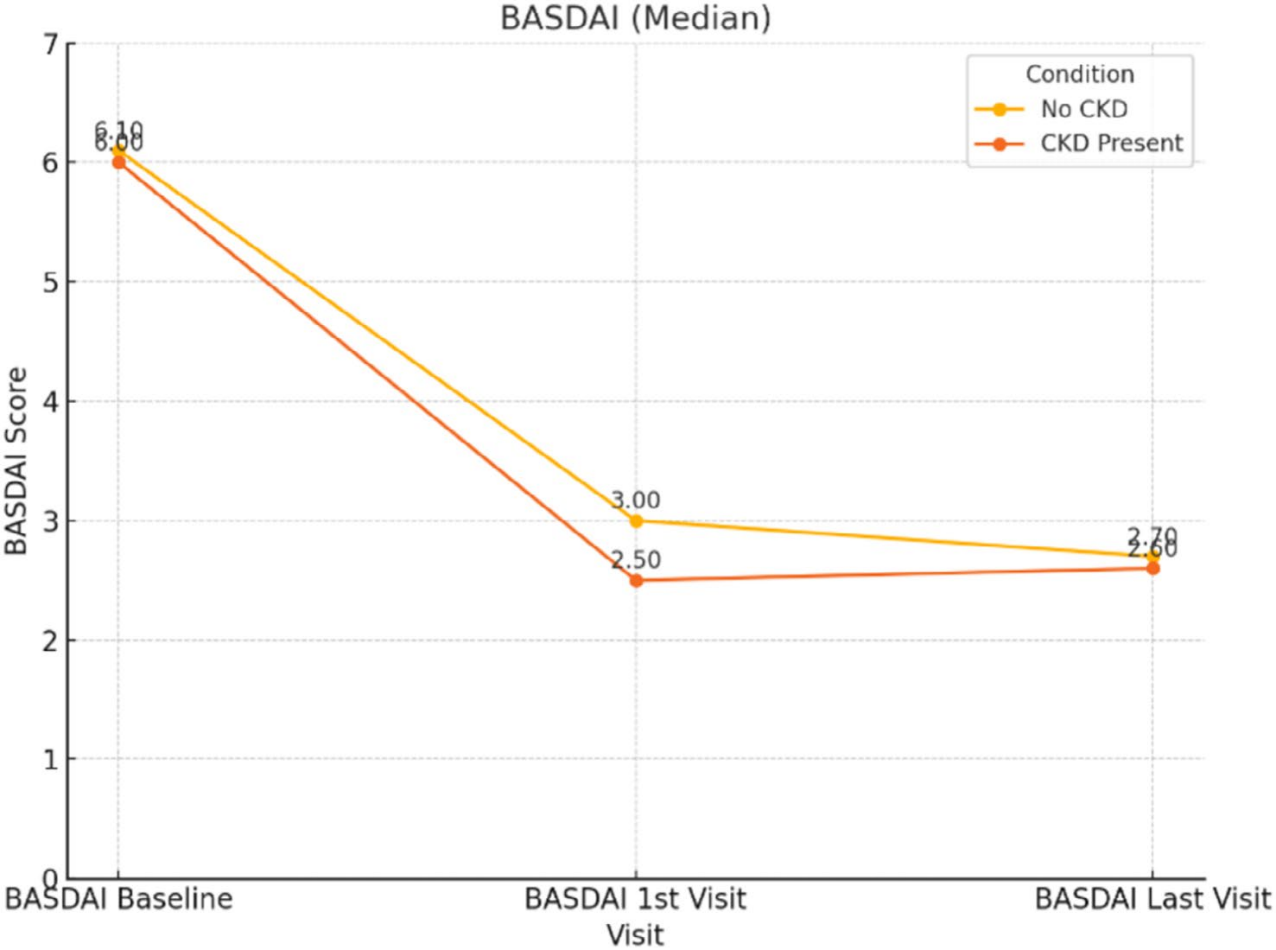
Baseline, first visit, and second visit BASDAI scores evaluated in CKD and non‐CKD patients.

**FIGURE 2:**
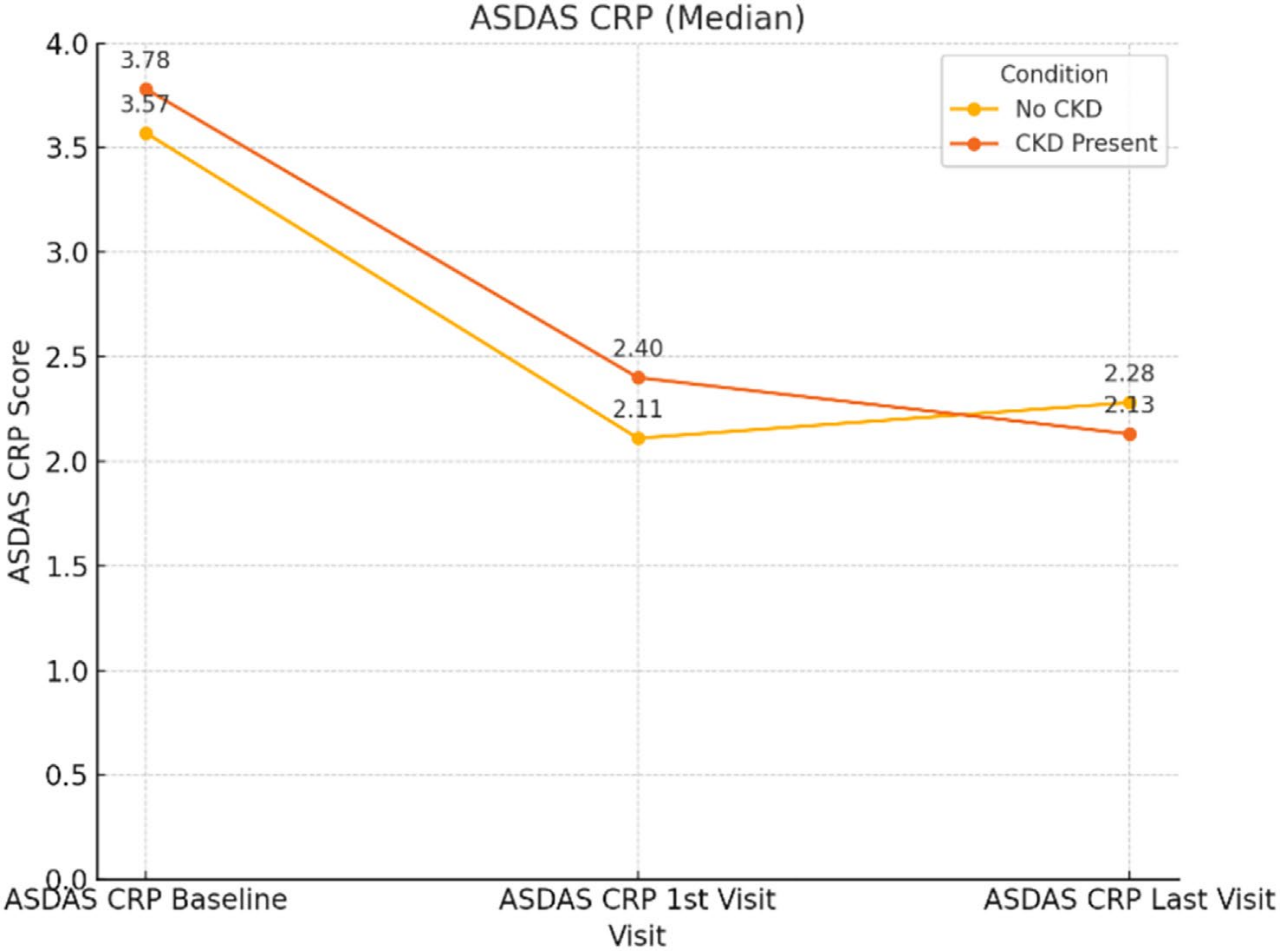
Baseline, first visit, and second visit ASDAS‐CRP scores evaluated in CKD and non‐CKD patients.

Baseline, first visit, and second visit BASDAI and ASDAS‐CRP scores were evaluated, and the corresponding graphs are presented (see Figures 1 and 2).

## 4 Discussion

This study demonstrated that biologic DMARDs are generally well tolerated in SpA patients with CKD, showing comparable drug retention times and adverse effect profiles to those without CKD. Despite concerns regarding renal impairment, no significant differences were observed in treatment discontinuation due to side effects between CKD and non‐CKD groups, supporting the safe use of bDMARDs in this population.

The introduction of bDMARDs has significantly improved the management of SpA and contributed to better patient quality of life [18]. Although the efficacy and safety of these agents have been well established in the general population, evidence regarding their use in specific subgroups—particularly individuals with renal impairment—remains limited [19, 20]. Renal dysfunction is a relevant concern in the context of SpA, as it may result from treatment‐related complications (e.g., due to DMARDs, NSAIDs, or MTX) or present as an extra‐articular manifestation of the disease [21]. Moreover, comorbid conditions including hypertension, diabetes mellitus, hyperlipidemia, and amyloidosis are also well‐established contributors to the development and progression of CKD.

Anti‐TNF agents play a central role in the treatment of SpA, owing to their well‐established efficacy and safety profiles. These biologics are primarily metabolized within lysosomes and are not significantly affected by renal function [22]. In the present study, no significant difference in drug retention time was observed between patients with and without CKD, suggesting that bDMARDs are well tolerated even in SpA patients with renal impairment. Among the biologic agents evaluated, etanercept was more frequently preferred in the CKD group, particularly as a first‐line treatment option. This preference may be attributed to etanercept's shorter half‐life, which may offer a safety advantage in patients with compromised renal function [23]. Similar to the current study's findings, Don et al. showed that the pharmacokinetics of etanercept in patients with end‐stage renal disease (ESRD) on hemodialysis (HD) were comparable to those in individuals with normal renal function, suggesting no need for dose adjustments in HD patients [24]. Fernández‐Nebro et al. found that anti‐TNF therapy, including infliximab and etanercept, was safe and effective in patients with RA, AS, and PsA who had amyloidosis [25]. Supporting these observations, Hueber et al. reported that TNF blockers—including infliximab, etanercept, and adalimumab—did not adversely affect renal function, even in patients undergoing HD [26]. Furthermore, large‐scale meta‐analyses involving up to 24,000 patients treated with anti‐TNF agents found no evidence of significant renal toxicity, further reinforcing the safety of these therapies in individuals with CKD [27]. Ustekinumab and secukinumab have demonstrated minimal renal toxicity in the literature. Although a single case of nephrotic syndrome has been reported with ustekinumab, it is generally considered safe for use in dialysis patients. Likewise, no nephrotoxic effects have been associated with secukinumab [28, 29]. Consistent with these findings, both agents were safely administered to CKD patients in the present study.

A substantial body of literature has documented the occurrence of infections during TNF inhibitor therapy. Numerous registry studies and meta‐analyses have reported a higher incidence of infections in patients receiving TNF‐α inhibitors compared to both the general population and those treated with csDMARDs [30]. In a more recent study, Don et al. reported that etanercept was well tolerated in their selected patient population, despite the well‐documented increased risk of infections among individuals undergoing dialysis [31]. Consistent with previous findings, the present study revealed no significant differences in infection‐related drug discontinuation between CKD and non‐CKD patients, despite the generally increased infection risk associated with CKD. Interestingly, allergic reactions were significantly more frequent in non‐CKD patients, which may be attributed to the relatively small sample size of the CKD group, immune dysregulation associated with CKD, or the more cautious selection of biologics by clinicians in this population. Similarly, although prior studies have shown a heightened risk of malignancy in CKD patients during both the predialysis and dialysis periods, the frequency of malignancy was comparable between the two groups in this cohort [32].

In both CKD and non‐CKD patients, a statistically significant reduction in BASFI, ASDAS‐CRP, and BASDAI scores was observed at the first and second follow‐up visits compared to baseline. These findings suggest that the treatment was effective even in patients with CKD, in line with previous reports in the literature [33].

When examining the use of csDMARDs prior to initiating biological therapy, leflunomide was found to be significantly more frequently used in patients with CKD [34]. This finding aligns with existing literature, which also suggests that leflunomide is considered safe in CKD. On the other hand, the significantly lower use of NSAIDs in patients with CKD is unsurprising, as current guidelines recommend avoiding NSAIDs in this patient population [35, 36]. No significant difference in MTX use was found prior to the initiation of bDMARD therapy, suggesting that MTX is relatively commonly used among SpA patients with CKD. This observation aligns with findings from a previous study, which reported that 65.6% of patients with inflammatory arthritis and CKD were treated with MTX [37]. Regular reassessment of csDMARD use according to renal function at each clinical visit, accompanied by appropriate dose adjustments or discontinuation when indicated, is essential for the effective management of patients with CKD [38].

Regarding comorbidities, it was not surprising that diabetes, hypertension, hyperlipidemia, and amyloidosis were more common in patients with CKD, as these factors are also frequently observed in the etiology of CKD as components of metabolic syndrome [39, 40]. Similarly, the higher prevalence of PsA in the CKD group may be explained by shared metabolic risk factors, including obesity, diabetes mellitus, hypertension, and hyperuricemia, which are common in both conditions [41]. Additionally, prolonged use of NSAIDs, which is often necessary for PsA symptom control, may contribute to renal impairment over time [42]. The relatively high prevalence of amyloidosis in the CKD group may also reflect the effects of long‐standing systemic inflammation and greater disease burden, as the study cohort was derived from tertiary referral centers that primarily manage more complex and treatment‐resistant SpA cases [43, 44]. Consistent with this, significantly elevated ESR and CRP levels were observed in CKD patients, likely reflecting the chronic inflammatory state associated with renal dysfunction [45]. Furthermore, patients in the CKD group had a higher mean age, longer disease duration, and increased BMI—all of which are established risk factors for the development and progression of CKD [46]. Age‐related decline in renal function and the adverse impact of obesity on renal health may also contribute to these findings [47].

This study has several limitations. First, its retrospective design and relatively small sample size may limit the generalizability of the findings. Additionally, data regarding bDMARD dosage and administration frequency were not available, and patients were not stratified according to the route of administration, which may have influenced drug retention outcomes. Renal biopsies were not performed in all patients, making it difficult to determine the exact etiology of CKD in certain cases. The study included only patients with pre‐existing CKD at the time of biologic therapy initiation, excluding those who developed CKD during or after treatment. As a result, potential dynamic effects of bDMARDs on renal function may have been overlooked. Furthermore, changes in CKD stage throughout the treatment period were not systematically evaluated, despite the known association between disease progression and increased complication risk. The analysis was also limited to adverse events that led to drug discontinuation, without addressing the full spectrum of potential side effects. In addition, treatment decisions may have been affected by national reimbursement policies and institutional restrictions, potentially influencing the observed patterns of biologic use.

Nonetheless, the study offers significant strengths. Its key strength lies in its pioneering approach as one of the earliest to examine the real‐world efficacy and safety of comprehensive bDMARD therapies. To the best of our knowledge, this study includes the largest cohort of SpA patients with renal involvement treated with TNF blockers reported in the literature to date.

In conclusion, this study offers preliminary evidence supporting the effective and safe use of bDMARDs in patients with SpA and CKD. Additional research is required to confirm these results, to establish optimal dosing strategies for many DMARDs, clarify the mechanisms driving the observed outcomes, and refine the use of bDMARDs in this specific patient population. Close monitoring of kidney function and vigilance for systemic drug toxicities are strongly advised for patients receiving these treatments.

## Author Contributions

The manuscript was evaluated and approved by all of the authors.

## Ethics Statement

The ethical approval for the TReasure database was obtained in May 2017 (KA‐17/058) and in October 2017 from the Ministry of Health (9318930414.03.01). The study received approval from the local ethics committee and was carried out in compliance with the principles outlined in the Declaration of Helsinki (1975/83).

## Consent

Written informed consent was obtained from all participants prior to their inclusion.

## Conflicts of Interest

The authors declare no conflicts of interest.

## Data Availability

The data that support the findings of this study are available upon request.
